# Vaccine Adverse Event Mining of Twitter Conversations: 2-Phase Classification Study

**DOI:** 10.2196/34305

**Published:** 2022-06-16

**Authors:** Sedigheh Khademi Habibabadi, Pari Delir Haghighi, Frada Burstein, Jim Buttery

**Affiliations:** 1 Centre for Health Analytics Melbourne Children’s Campus Melbourne Australia; 2 Department of General Practice University of Melbourne Melbourne Australia; 3 Department of Human-Centred Computing, Faculty of Information Technology, Monash University Melbourne Australia; 4 Department of Paediatrics University of Melbourne Melbourne Australia

**Keywords:** immunization, vaccines, natural language processing, vaccine adverse effects, vaccine safety, social media, Twitter, machine learning

## Abstract

**Background:**

Traditional monitoring for adverse events following immunization (AEFI) relies on various established reporting systems, where there is inevitable lag between an AEFI occurring and its potential reporting and subsequent processing of reports. AEFI safety signal detection strives to detect AEFI as early as possible, ideally close to real time. Monitoring social media data holds promise as a resource for this.

**Objective:**

The primary aim of this study is to investigate the utility of monitoring social media for gaining early insights into vaccine safety issues, by extracting vaccine adverse event mentions (VAEMs) from Twitter, using natural language processing techniques. The secondary aims are to document the natural language processing techniques used and identify the most effective of them for identifying tweets that contain VAEM, with a view to define an approach that might be applicable to other similar social media surveillance tasks.

**Methods:**

A VAEM-Mine method was developed that combines topic modeling with classification techniques to extract maximal VAEM posts from a vaccine-related Twitter stream, with high degree of confidence. The approach does not require a targeted search for specific vaccine reaction–indicative words, but instead, identifies VAEM posts according to their language structure.

**Results:**

The VAEM-Mine method isolated 8992 VAEMs from 811,010 vaccine-related Twitter posts and achieved an *F*_1_ score of 0.91 in the classification phase.

**Conclusions:**

Social media can assist with the detection of vaccine safety signals as a valuable complementary source for monitoring mentions of vaccine adverse events. A social media–based VAEM data stream can be assessed for changes to detect possible emerging vaccine safety signals, helping to address the well-recognized limitations of passive reporting systems, including lack of timeliness and underreporting.

## Introduction

### Background

Vaccines belong to the broad category of medicines, in a subcategory known as *biologicals* [1]. Unlike medicines that are prescribed to limited populations as a course of *treatment* for a disease, vaccines are given to both healthy and vulnerable populations at large, sometimes over a short period, to enhance their immune systems’ ability to combat a pathogen. In contrast to those who are taking a medicine to help to cure a disease or to treat unwanted symptoms, most people receiving a vaccine are not ill. Therefore, there is a deferred individual benefit to taking a vaccine, and, consequently, a very low acceptance of risk regarding vaccines [2]. In addition, the pathophysiology of vaccine-related adverse events is not as well defined as those of adverse drug reactions—a reaction triggered by a vaccine could be caused by any of its multiple ingredients, its underlying technology (eg, messenger RNA–based vs protein-based delivery), or even an error in administration [3]—and some people are particularly prone to reacting to vaccine ingredients [4]. Furthermore, a vaccine’s *time to market* may be curtailed, such as has occurred during the COVID-19 pandemic, and so provide less opportunities for studying potential vaccine side effects over a large population for a long time.

Vaccine safety relies upon rigorous compliance to development and manufacturing standards, well conducted clinical trials, thorough assessment, licensing, control, and administration of vaccines. Postlicensure vaccine safety surveillance is a key component of ensuring vaccine safety [5] and continues in a variety of forms after regulatory approval or emergency use authorization. It is the primary mechanism to identify serious or rare adverse events following immunization (AEFI) that are unlikely to have been exposed by prelicensure trials, and it allows surveillance in populations that were unable to be included in the trials [6]. Identification of minor AEFI is potentially as important as those of severe adverse events, as minor AEFI may act as a surrogate warning for more severe sequelae (eg, increased rates of fever may be a marker for increased febrile seizures [7])—that is, increased incidences of even minor events could indicate larger problems.

Traditional passive (spontaneous) surveillance systems, where a voluntary reporting of AEFI is made by individuals or by their treating health professionals, are the main method of vaccine safety monitoring and have proven to be useful in early detection of vaccine-related and drug-related safety issues [8,9]. Although these systems are the backbone of drug safety monitoring, they suffer from major disadvantages, including underreporting, incomplete data, and time lag between an event happening and subsequent reporting of it [10]. Active surveillance systems survey vaccine recipients and vaccine administrators to determine the outcomes of recent vaccinations, irrespective of any AEFI experience. Increasingly, alternate data sources are being added to surveillance systems, as they offer the potential to capture timely and additional measurements of the quantity of possible adverse events.

Extensive use of social media has provided a platform for sharing and seeking health-related information. Social media data have consequently become a widely used source of data for public health research [11]. In comparison with established traditional surveillance systems, social media monitoring is inexpensive and near to real time and covers large populations [12], thus offering an easily accessible wide-ranging data source for tracking emerging trends—which may be unavailable or less noticeable in data gathered by traditional reporting systems [13].

Many researchers have used social media as a pharmacovigilance source [14]. However, there is relative deficit in the use of social media for AEFI detection. Many investigations of vaccine and vaccination-related social media posts are related to sentiments, attitudes, and opinions [15-21]. Studies on using social media for detection of adverse drug reaction have included vaccine-related words in keyword searches used for collecting data. An example is an annotated data set of tweets containing 250 drug-related keywords, including *vaccine*, for over a period of 4 months [22]. We downloaded and assessed these data sets, but they did not contain any AEFI data. A total of 2 recent studies have focused on detecting influenza [23] and COVID-19 [24] vaccine adverse events from Twitter. However, the emphasis of both these studies were on identifying specific vaccine adverse events using a lexicon of adverse reactions.

### Objectives

In this paper, we use the term *vaccine adverse event mention* (VAEM) to refer to *any* vaccine-related personal health mention, that is, VAEMs are conversations that contain personal health mentions in a vaccine context. This distinguishes VAEM from the AEFI and adverse drug reaction signals used in previous studies on the use of social media for vaccine and drug reaction surveillance, as these are searching for specific adverse vaccine events and drug reactions.

Although vaccine safety surveillance systems monitor for unexpected, rare, and late-onset events, they also aim to observe changes in the rate of known and expected events, because “while rare but particularly serious events can be detected through review of each individual report or active surveillance, an increased incidence in a more common AEFI is often more difficult to detect, and has been described as akin to ‘finding a needle in the haystack’” [13]. VAEM are conversations, ideally gathered in volume, that contain information that may be the common AEFI that are so elusive to traditional reporting, while also allowing the detection of previously unknown severe events.

This paper presents the VAEM-Mine method, which encapsulates the workflow and techniques required to enable detection of VAEM by applying natural language processing techniques to a relatively unfocused social media stream, consisting of any vaccine-related Twitter conversation. The VAEM-Mine method detects likely VAEM based on their characteristics of being *personal health mentions* in a vaccination context. VAEM-Mine has 2 components—a topic modeling process that initially detects and filters for VAEM (described in a previous publication [25]) and a classification task that accurately identifies VAEM in the filtered data—which is described in detail in this paper.

## Methods

### Ethics Approval

Ethics approval for this study was granted by Monash University Human Research Ethics Committee (project ID 11767).

### Data Collection

The Twitter application program interface was used to collect English tweets with search terms *vaccination, vaccinations, vaccine, vaccines, vax, vaxx, vaxine, vaccinated, vaccinated, flushot,* and *flu shot*. These were general terms that were designed to collect a broadly representative sample of vaccine-related conversations. We included *flu shot* as a keyword because we found that this was most often used, rather than the term *flu vaccine*, whereas other vaccines were usually mentioned in conjunction with the word *vaccine*—and thus, for them, we only needed to search for *vaccine* keywords. Upon examining the downloaded data for specific vaccine names, we found more records mentioning other vaccines than those mentioning the influenza vaccine. No specific reaction mentions were used.

A total of 400,000 tweets were initially collected across 5 months, from February 7, 2018, to June 7, 2018, which were used for an initial training and evaluation of topic models and classifiers. An additional 411,010 tweets were collected from August 9, 2018, to July 20, 2019, which were used to verify the trained topic models and classifiers and to train more powerful classifiers. The resulting data consisted of a total of 811,010 tweets and a daily average of 2906 tweets.

The data were prepared by removing URLs and by converting to lower case. Duplicates were removed based on tweet ID and text. Other preparation included removing hashtags, usernames, punctuation, and numbers. Tweets with <5 words were removed. N-grams were created for topic modeling; preparation for classification is explained in the following section. The final cleaned tweets were 82.21% (328,822/400,000) of the initial collection and 87.48% (359,535/411,010) of the second collection—a total of 688,357.

Table 1 illustrates a sample of tweets that mention receiving vaccinations or vaccines. The first 3 examples contain genuine VAEM, but the others do not—even when the language is similar. Our goal was to first isolate the most likely records describing personal experiences of vaccination and then to refine that selection to those that are genuine adverse reaction mentions.

**Table 1:** Sample of vaccine-related tweets.

Tweet	Type
“Aw wtf my poor arm is dead af from my flu shot.”	VAEM^a^
“Cannot lie on belly, baby gets squished; cannot lie on back, baby squishes; cannot lie on right side, i get heartburn; cannot lie on left side, vax arm is sore; let the third trimester moaning begin!”	VAEM
“2 people recently, including my 88yo father, had flu shot and really bad reaction afterwards. both said it was probably as bad as getting the flu!!! flu2018 maybe undercooked the vaccine.”	VAEM
“I got vaccinated as a kid. As a result, I'm now starting to gray and bald. My balding got so bad I had to shave my head. I've also gained weight. Because of vaccines I've started aging instead of dying as a baby.”	Non-VAEM
“Urgent vaccination plea after measles outbreak in West Yorkshire.”	Non-VAEM
“Researchers are developing a personalized vaccine which they hope could tackle ovarian cancer.”	Non-VAEM

^a^VAEM: vaccine adverse event mention.

The topic modeling showed that VAEM and similar personal health mentions were a distinct topic (among 13 vaccine-related topics), and therefore, that topic models could be used to filter for the tweets that were most similar to VAEM. Taking tweets from only that topic meant that relatively homogenous data sets could be created for labeling and subsequent training of classifiers. The use of topic modeling for filtering data before classification was adopted as a core component of the VAEM-Mine method. A previous publication [25] described the process of choosing the best performing topic models for the method, including a detailed description of the scoring method used to identify the best models.

### Classification

#### Overview

As described in the previous section, data were collected in 2 phases. Topic models were trained on the first-phase data and were used to filter that data and the subsequent second-phase data into likely VAEM-containing data sets, which were then used for classification. Classifiers were trained and assessed with the filtered first-phase data set and the combined (filtered) first-phase and second-phase data sets. The following section describes the creation of these data sets; the subsequent section describes the classifiers.

#### Classification Data Sets

The original prepared (cleaned) data collections of 328,822 and 359,535 tweets were reduced, by applying topic model–based filtering, to data sets containing 18,801 (5.72%) and 80,372 (22.35%) tweets that were more likely to contain VAEMs—a total of 99,173 tweets, which was only 14.41% (99,173/688,357) of the total original cleaned data.

Therefore, filtering eliminated approximately 85.59% (589,184/688,357) of the data, which did not contain any significant numbers of VAEM. These more VAEM-focused data sets were binary labeled by the author (SKH), as either VAEM or non-VAEM. All the labels were verified by the domain expert. Although only 10.07% (9991/99,173) of the tweets were identified as VAEM, this was a considerably better proportion of VAEM compared with the original cleaned data, which contained VAEM in only 1.45% (9991/688,357) of the tweets.

Balanced data sets of 18.72% (3519/18,801) and 19.57% (15,730/80,372) of the tweets were created from these imbalanced data sets together with holdout test data sets—these were an imbalanced test set of 3.27% (614/18,801) of the tweets and a balanced test set of 1.03% (828/80,372) of the tweets. The main data sets were named *Phase-One* and *Phase-Two* data sets, and the test data sets were referred to as *Phase-One Test* and *Phase-Two Test* data sets.

The imbalanced Phase-One Test data set of 3.27% (614/18,801) of the tweets were obtained from Victoria, Australia, in the period preceding and during the 2018 influenza immunization period. These tweets were assembled to enable comparison of tweet trends with statistics from the Australian Victorian vaccine authority, Surveillance of Adverse Events Following Vaccination In the Community. With 90 VAEM and 524 non-VAEM, the test set was imbalanced but reflected how the data were obtained through the topic model filtering process, without any subsequent balancing. The Phase-One Test data set was used as a benchmark throughout the classification testing. The data sets (Table 2) were combined to retrain classifiers and train transformer-based classifiers—becoming a C*ombined* data set of 19,249 tweets and a *Combined Test* data set of 1442 tweets. The training data were split into training and validation data with a 75:25 ratio.

**Table 2:** Data set numbers.

Stage	Phase-One data, n (%)	Phase-Two data, n (%)	Total, n
Topic modeling	328,822 (47.77)	359,535 (52.23)	688,357
Filtering out by topic modeling	−310,021 (52.62)	−279,163 (47.38)	−589,184
After topic modeling	18,801 (18.96)	80,372 (81.04)	99,173
Filtering out by data preparation and balancing	−14,668 (18.69)	−63,814 (81.31)	−78,482
For classification training	4133 (19.97)	16,558 (80.03)	20,691
For training and validation	3519 (18.28)	15,730 (81.72)	19,249
For testing	614 (42.58)	828 (57.42)	1442

#### Classifiers

Our default data approach with traditional models (ie, not neural network–based) was *bag-of-words* [26], represented via compressed sparse matrices. We used SKLearn (Scikit-learn) [27] vectorizing libraries such as TfidfTransformer [28] for tokenizing lowercase text for the standard classifiers. A grid or random search was used to ascertain the best combinations of vectorizer, removal of stop words and numbers, and n-grams. The neural networks used dense word embedding vectors via a Word2Vec skip-gram corpus [29] for Convolutional Neural Networks (CNNs) and Long Short-Term Memories (LSTMs), and the Word2Vec corpus used Gensim library functions [30] using all the Twitter data. The transformer models used byte-pair-encoding [31]; the byte-pair-encoding tokens were derived only from the filtered texts we had retained from topic modeling. The classifiers are listed in Table 3, and details of their definitions and parameters are listed in Multimedia Appendix 1.

**Table 3:** List of classifiers.

Models	Library or GitHub source
LR CV^a^	sklearn.linear_model [32]
SGD^b^ Classifier	sklearn.linear_model [32]
Linear SVC^c^	sklearn.svm.SVC [33]
RF^d^	sklearn.ensemble [34]
Extra Trees	sklearn.ensemble [34]
Multinomial NB^e^	sklearn.naive_bayes [35]
NB SVM^f^ (combined NB and Linear SVM)	GitHub Joshua-Chin/nbsvm [36]
XGBoost^g^	GitHub dmlc/xgboost [37]
Ensemble (NB SVM, LR CV, SGD, Linear SVC, and RF)	Majority voting [38]
CNN,^h^ LSTM,^i^ BiLSTM,^j^ GRU,^k^ BiGRU,^l^ CNN-BiLSTM, and CNN-BiGRU	Pytorch [39], RaRe-Technologies [30], Shawn1993 [40], and bamtercelboo [41]
RoBERTa,^m^ RoBERTa Large, BERT,^n^ XLNet,^o^ XLNet Large, and XLM^p^	Pytorch; huggingface transformers [42]

^a^LR CV: Logistic Regression Cross Validation.

^b^SGD: Stochastic Gradient Descent.

^c^SVC: Support Vector Classification.

^d^RF: Random Forest.

^e^NB: Naïve Bayes.

^f^SVM: Support Vector Machine.

^g^XGBoost: Extreme Gradient Boosting.

^h^CNN: Convolutional Neural Network.

^i^LSTM: Long Short-Term Memory.

^j^BiLSTM: Bidirectional LSTM.

^k^GRU: Gated Recurrent Unit.

^l^BiGRU: Bidirectional Gated Recurrent Unit.

^m^RoBERTa: Robustly Optimized Bidirectional Encoder Representations Pretraining Approach.

^n^BERT: Bidirectional Encoder Representations.

^o^XLNet: Generalized Autoregressive Pretraining for Language Understanding.

^p^XLM: Cross-Lingual Language Model.

### VAEM-Mine Method

**Figure 1:**
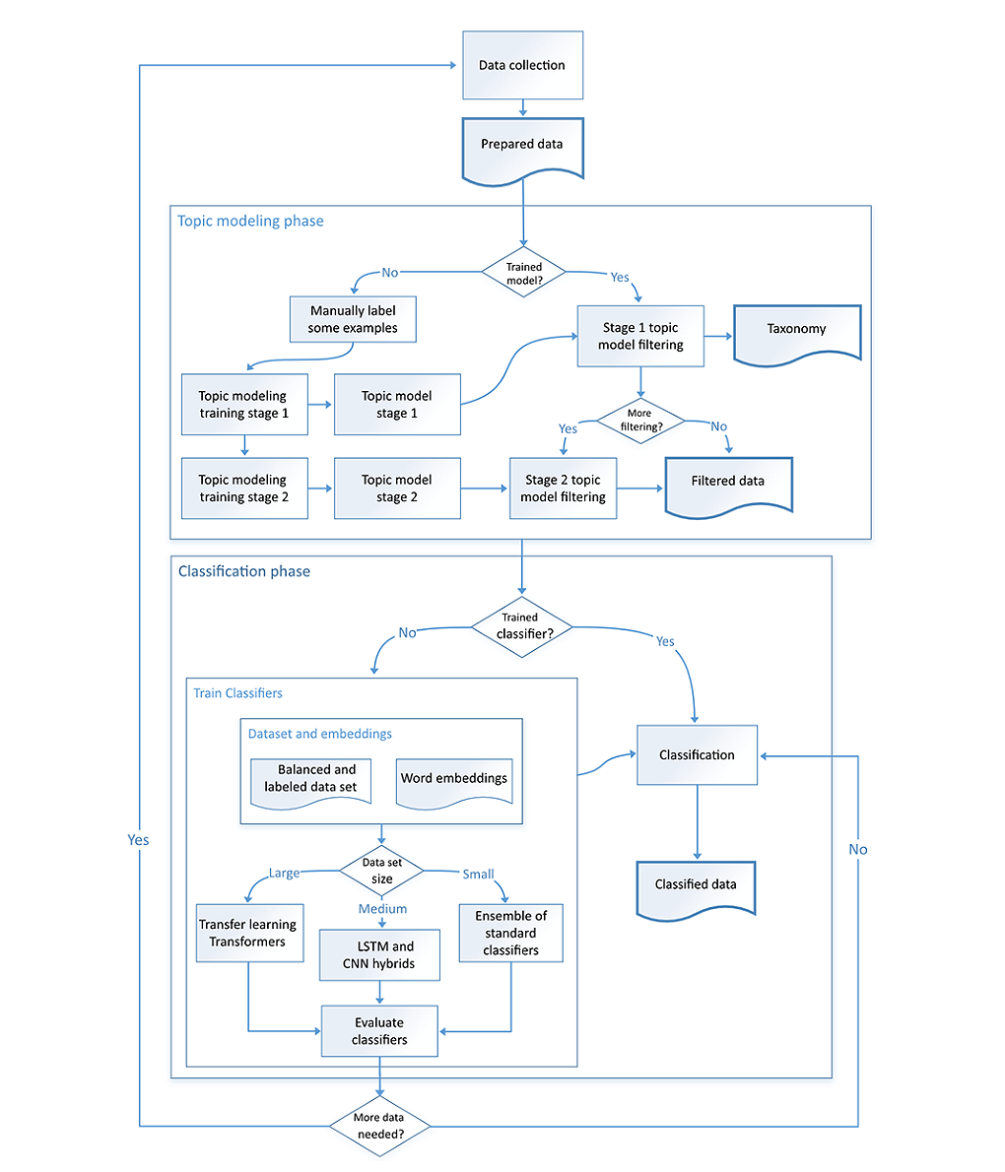
The vaccine adverse event mention–mine method. CNN: Convolutional Neural Network; LSTM: Long Short-Term Memory.

The classification models were the final component of a pipeline named the VAEM-Mine method (Figure 1), consisting of processes that started with data collection and cleaning, followed by processing through topic models to filter for data that were as close as possible to the VAEM, and then, a focused binary classification approach for isolating VAEM.

The method included decision points to determine the appropriate direction, either the training process or the application of the trained models to incoming data. At the beginning of the topic modeling phase, a trained model did not exist; thus, the work of training the topic models began. The first step was to label some examples of the subject of interest (in this case, VAEM) and additional examples of other subjects. This enabled the application of a topic modeling scoring, which measured how the VAEM-label of interest was distributed in the topics, compared with other labeled topics. A topic model was considered to score well if the VAEM were concentrated in only a few topics, and ideally in only 1 topic, with minimum data belonging to the other labels. Further refinement of the data was possible by a second stage of topic modeling on the data obtained from the top model of the first stage. The second stage identified topics that had a high ratio of VAEM to other subjects in the texts, but at the expense of losing some texts containing VAEM. Having trained the models, they could be applied to filter the incoming data, and it was up to the user whether they take only the output of the best topic (or topics) of the first-stage topic model or further refine the data by taking it from selected topics of the second-stage topic model. The topics of the first stage of topic modeling were also potentially useful to obtain a domain taxonomy.

The filtered data were handled by the classification phase, which also had the decision point for either training classifiers or using trained classifiers. When training, the choice of classifiers should relate to the quantity of available data, and if results are not as expected, a decision may be made to obtain more data. The method required the incoming filtered data to be labeled for the creation of data sets suitable to train the classifiers. It additionally required the creation of domain-specific embeddings. The VAEM-Mine method can be adopted as a workflow to tackle any similar task of identifying personal health mentions.

## Results

### Classification Analysis

Classification training and evaluation was conducted twice; first, with the filtered data that were obtained from applying topic modeling to the initial phase of data collection and then, with the data obtained through topic model filtering over all the collected data. The following sections describe these as Phase-One and Phase-Two classification.

#### Phase-One Classification

The first phase of classification experiments used a training set of 2639 records, a validation set of 880 records, and the imbalanced holdout Phase-One Test data set of 614 tweets. The *F*_1_ scores for the models evaluated in this phase are listed in Table 4.

**Table 4:** Phase-One F1 scores.

Model	Validation	Imbalanced test	Balanced test	Combined test
CNN^a^-BiGRU^b^	0.842	0.762	0.846	0.825
BERT^c^	N/A^d^	0.767	0.841	0.824
BiGRU	0.807	0.793	0.828	0.822
CNN–LSTM^e^	0.805	0.777	0.815	0.808
BiLSTM^f^	0.815	0.807	0.807	0.807
GRU^g^	0.820	0.730	0.822	0.804
CNN-BiLSTM	0.816	0.766	0.810	0.802
CNN	0.816	0.787	0.800	0.798
LSTM	0.796	0.767	0.803	0.796
Ensemble	0.815	0.726	0.829	0.810
Logistic Regression CV^h^	0.812	0.730	0.820	0.803
Linear SVC^i^	0.814	0.693	0.824	0.797
SGD^j^	0.805	0.636	0.825	0.785
Naïve Bayes SVM^k^	0.792	0.767	0.789	0.785
Random Forest	0.814	0.694	0.801	0.779
Extra Trees	0.833	0.688	0.801	0.777
XGBoost^l^	0.811	0.704	0.791	0.774
Naïve Bayes	0.798	0.605	0.799	0.756

^a^CNN: Convolutional Neural Network.

^b^BiGRU: Bidirectional Gated Recurrent Unit.

^c^BERT: Bidirectional Encoder Representations.

^d^N/A: not applicable.

^e^LSTM: Long Short-Term Memory.

^f^BiLSTM: Bidirectional Long Short-Term Memory.

^g^GRU: Gated Recurrent Unit.

^h^CV: Cross Validation.

^i^SVC: Support Vector Classification.

^j^SGD: Stochastic Gradient Descent.

^k^SVM: Support Vector Machine.

^l^XGBoost: Extreme Gradient Boosting.

Table 4 includes subsequent tests of the models against the Phase-Two *Balanced test* data set and a *Combined Test* data set that uses all the test data. *F*_1_ scores were measured for the positive, VAEM class, rather than for both classes. The models are arranged in order of the best *F*_1_ score over the test data sets; validation scores are also included, where available. Validation *F*_1_ scores are not available for models using transfer learning—they used a cross-validation approach, and thus, were given combined training and validation data and were evaluated only against test data sets.

The Ensemble model shown in the middle of Table 4 was scored based on a maximum voting of the predictions of 5 traditional classifiers on the test data set—consisting of the Naïve Bayes Support Vector Machine, Linear Regression Cross Validation, Stochastic Gradient Descent, Linear Support Vector Classification, and Random Forest classifiers. It had the overall best score among the traditional classifiers on the large test data.

All the deep learning models outperformed the best traditional classifier on the *Imbalanced Test* data set, by at least 6% and almost as much as 10%—the improvement was mostly owing to great capacity to correctly distinguish non–VAEM-related tweets, and thus obtain a greater precision. However, when evaluated against the *Balanced* and *Combined Test* sets, the results differed—here, the traditional classifiers outperformed many of the deep learning models, especially the Ensemble, which was only surpassed by the top 3 deep learning models.

#### Phase-Two Classification

The second phase of classification used 5 times as many records to train the models, by combining the 3519 training records from the first phase with another 15,730 records, resulting in a total of 19,249. Phase Two also introduced a large, more balanced test data set of 828 records. The greater amount of data allowed a proper assessment of neural networks, but it also improved model performance across the board (Table 5). The *imbalanced change* and *combined change* columns show the percentage increase in the models’ *F*_1_ score over the *Imbalanced Test* and *Combined Test* data sets, compared with their Phase-One equivalents.

There was a much greater consistency of scoring over all the test data sets, and the top models scored best over all the test data sets. The highest score was from the Robustly Optimized Bidirectional Encoder Representations Pretraining Approach (RoBERTa) Large Transformer model, with an *F*_1_ score of 0.919 on the Imbalanced data set; the standard RoBERTa model was placed second.

One of the most noteworthy effects of having more data was that the previously strong combinations of CNN with Bidirectional Gated Recurrent Unit and Bidirectional LSTM models were surpassed by the LSTM on the *Imbalanced Test* data set, both when combined with a CNN but most significantly as a stand-alone model. The LSTM in fifth position on the imbalanced test scoring was only 2.5% behind the score of the RoBERTa Large model. One can fairly conclude that a CNN or hybrid CNN approach performs well when limited data are available but will likely be surpassed by architectures designed for sequential language processing as more data become available.

A detailed analysis of the classifiers’ performance is provided in Multimedia Appendix 2.

**Table 5:** Phase-Two F1 scores.

Model	Validation	Imbalanced test	Balanced test	Combined test	Imbalanced change, %	Combined change, %
RoBERTa^a^ Large	N/A^b^	0.919	0.908	0.910	—^c^	—
RoBERTa	N/A	0.901	0.905	0.904	—	—
XLNet^d^ Large	N/A	0.884	0.906	0.902	—	—
XLNet	N/A	0.870	0.903	0.897	—	—
XLM^e^	N/A	0.910	0.894	0.897	—	—
BERT^f^	N/A	0.863	0.892	0.887	12.6	7.7
BiGRU^g^	0.877	0.855	0.896	0.890	7.9	8.2
CNN^h^-BiGRU	0.874	0.849	0.890	0.884	11.4	7.1
LSTM^i^	0.866	0.875	0.879	0.878	14.1	10.3
CNN-LSTM	0.866	0.862	0.876	0.873	10.9	8.1
BiLSTM^j^	0.872	0.847	0.884	0.878	5	8.8
GRU^k^	0.869	0.825	0.876	0.868	13.1	7.9
CNN-BiLSTM	0.872	0.824	0.879	0.871	7.6	8.6
CNN	0.864	0.805	0.866	0.856	2.4	7.2
Ensemble	0.870	0.818	0.874	0.865	12.6	6.8
Logistic RCV^l^	0.866	0.807	0.873	0.861	10.5	7.3
SGD^m^	0.865	0.806	0.873	0.861	26.7	9.7
Linear SVC^n^	0.864	0.802	0.869	0.857	15.7	7.5
Random Forest	0.857	0.796	0.864	0.853	14.7	9.5
Extra Trees	0.857	0.789	0.862	0.849	14.7	9.2
NB^o^ SVM^p^	0.838	0.798	0.838	0.832	3.9	5.9
XGBoost^q^	0.845	0.714	0.854	0.831	1.3	7.4
NB	0.835	0.735	0.841	0.822	21.5	8.7

^a^RoBERTa: Robustly Optimized Bidirectional Encoder Representations Pretraining Approach.

^b^N/A: not applicable.

^c^Change calculation was not performed because no previous figures existed.

^d^XLNet: Generalized Autoregressive Pretraining for Language Understanding.

^e^XLM: Cross-Lingual Language Model.

^f^BERT: Bidirectional Encoder Representations.

^g^BiGRU: Bidirectional Gated Recurrent Unit.

^h^CNN: Convolutional Neural Network.

^i^LSTM: Long Short-Term Memory.

^j^BiLSTM: Bidirectional Long Short-Term Memory.

^k^GRU: Gated Recurrent Unit.

^l^RCV: Regression Cross Validation.

^m^SGD: Stochastic Gradient Descent.

^n^SVC: Support Vector Classification.

^o^NB: Naïve Bayes.

^p^SVM: Support Vector Machine.

^q^XGBoost: eXtreme Gradient Boosting.

### VAEM-Mine Method Performance

Here, we assess the overall effectiveness of the method, regarding the quantities of tweets having VAEMs that were progressively filtered out by the method. The values presented are the total numbers of tweets collected and processed via the method, with estimates where appropriate.

#### Topic Modeling Phase

Table 6 depicts the numbers obtained from after data collection to the completion of the topic modeling. From the original 811,010 records, 122,653 (15.12%) records were removed by data cleaning, and topic modeling was used to process 688,357 (84.87%) records. Stage 1 of topic modeling filtered out 82.86% (570,383/688,357) of the records to retain 17.14% (117,974/688,357) of the records likely to contain VAEM. The data were approximately 14.55% (117,974/811,010) of the original total and contained >99% of all the available VAEM (Multimedia Appendix 3).

**Table 6:** Summary of topic modeling counts (N=811,010).

Steps	Counts, n (% of initial data)
Tweets collected	811,010 (100)
Cleaned	–122,653 (–15.12)
Tweets after cleaning	688,357 (84.88)
Discarded (stage 1)	–570,383 (–70.33)
Tweets after stage 1	117,974 (14.55)
Discarded (stage 2)	–19,083 (–2.35)
Tweets after stage 2^a,b^	98,891 (12.19)

^a^Stage 2 proportions—non–vaccine adverse event mention: 88,900 and vaccine adverse event mention: 9991 (10.10% of stage 2 data; 1.45% of tweets after cleaning; 1.23% of initial data).

^b^Vaccine adverse event mention proportions—in other stage 2 topics: 2367 and in best stage 2 topic: 7624 (76.31% of vaccine adverse event mention).

To prepare for the first round of classification, additional 19,083 records were discarded—those which were not in the top 3 topics of the stage 2 topic model. Subsequent labeling of the discarded topic most likely to contain VAEM (based on the distribution of topic model labels) showed only 1.49% (94/6274) of VAEM in the data, which was approximately 5.15% (94/1826) of the VAEM in the first round.

For the second round of classification, all the records that were identified as likely VAEM by the topic model were retained. The resulting 12.19% (98,891/811,010) records retained over both rounds of topic modeling were labeled, and VAEM were found to be 10.10% (9991/98,891) of the retained data. The stage 2 topic models’ topic numbers were assessed, and it was found that the best stage 2 topic of 14,498 tweets contained 76.31% (7624/9991) of the retained VAEM, and there were approximately 11.10% (7624/6874) more VAEM than non-VAEM in the topic.

From these figures, we conclude that topic modeling is an effective filtering mechanism, as it identified approximately all the VAEM, while removing a lot of unwanted data. The filtered data were more manageable for labeling for classification than it would have otherwise been, and if needed, the filtered output of the stage 2 topic model can be used as it is, with the understanding that it discards some VAEM and still contains a small but similar number of non-VAEM. However, as discussed previously, classification is a more precise final step to obtain VAEM from the filtered records.

#### Classification Phase

To assess classifier effectiveness regarding the total data, the recall and precision of the best classifier, the RoBERTa Large model, were applied to the total VAEM to obtain an *estimate* of its performance on the total VAEM. These were a precision score of 0.874 and a recall score of 0.948 for the combined test data:

1. Applying the recall score of 0.948 to the total 9991 VAEM-containing tweets, we estimate that 94.81% (9472/9991) of the VAEM tweets would be correctly classified and 5.19% (519/9991) of the VAEM would be missed.
2. We find that 1.54% (1370/88,900) of the non-VAEM tweets would be added to the 9472 tweets to match to the precision score of 0.874 (9472/10,842).
3. These results of 94.81% (9472/9991) of VAEM together with 1.54% (1370/88,900) of the non-VAEM in the predicted positive class were clearly superior to those obtained with the best topic of stage 2 topic modeling, where we saw the proportion of VAEM in the best topic was 76.31% (7624/9991) and the almost equal number of non-VAEM in the topic was approximately 7.70% (6847/88,900) of the non-VAEM.

#### Combined Topic Modeling and Classification Effectiveness

By measuring the combined effectiveness of topic modeling and classification, the following results are estimated:

1. As explained in Multimedia Appendix 3, counts of VAEM identified via topic modelling were estimated to be 99% of all likely VAEM; therefore, with 99% being represented as a count of 9991 VAEM, it is estimated that 10,090 VAEM originally existed.
2. A total of 8992 VAEM are estimated to be identified via the combined effects of cleaning, topic modelling, and classification from the original 811,010 records, being at least 89.11% (8992/10,090) of all likely VAEM and 1.11% (8992/811,010) of the original data.
  A total of 98.89% (802,018/811,010) of the data were eliminated through cleaning, topic modeling, and classification.
  Totally, around 11% (1098/10,090) of the VAEM were also eliminated during this processing; the attrition is a consequence of the filtering and classification required to capture the estimated 89.12% (8992/10,090).
3. Overall, 98.89% (802,018/811,010) of data were eliminated as not containing VAEM, with a very small amount misidentified, to identify 1.11% (8992/811,010) of the data as having VAEM, with 90% success.

The results indicate that the combined approach of topic modeling followed by classification effectively identifies and isolates VAEMs from approximately all other vaccine-related Twitter posts. The VAEM-Mine method enables us to identify the most effective topic models and classifiers for the core task of isolating VAEM. In particular, the key to the method’s success is the topic modeling phase, which drastically reduces the amount of irrelevant data and thus delivers manageable data to the classification phase. As natural language processing technologies improve and new topic models and classifiers can be introduced, we assume that even these results will improve.

## Discussion

The key objective of this study was to contribute to research on vaccine safety surveillance, by illustrating that social media monitoring has the potential to augment existing surveillance systems. We have demonstrated a topic modeling and classification VAEM-Mine method for identifying VAEM with high degree of sensitivity and specificity following vaccination.

### Principal Findings

The VAEM-Mine method approached the problem of finding sparse VAEMs by using topic modeling followed by classification. Topic modeling identified texts based on their semantic and syntactic nature. Then, it was used to extract those tweets that predominantly describe personal health issues in relation to vaccines. Classification identified VAEMs from the filtered texts with high degree of accuracy. Neither of the machine learning components were explicitly trained on specific reaction keywords, instead they identified texts owing to their innate capacity to detect patterns in language structure.

Other studies on detecting influenza [23] and COVID-19 [24] have required purpose-built machine learning classifiers that identify specific adverse event reactions from tweets. Their classifiers were trained to identify known reaction keywords derived from medical databases. Our approach relies on language features of the tweets to elicit the likely cohort and the power of modern transformer classifiers to determine the true signals. By tackling the problem of finding adverse events through the lens of the language used in personal health mentions, we conclude that social media can provide a wealth of useful data.

The VAEM-Mine method has significant capability to successively isolate VAEMs from the massive amount of other vaccine-related Twitter posts. The topic modeling phase could isolate up to 99.02% (9991/10,090 [estimated]) of the Twitter posts that contained VAEM. The data identified by Stage 1 topic modelling as likely containing VAEM were only 14.55% (117,974/811,010) of the original data, thereby eliminating 85.45% (693,306/811,010) of mostly irrelevant posts. The classification phase identified 8992 (90%) of the 9991 VAEM with an *F*_1_ score of 0.91. The combination of topic modelling and classification resulted in the identification of 89.12% (8992/10,090 [estimated]) of the VAEM.

Training the topic modeling component of the method is enabled by identifying the most effective topic models by using *F*_1_ scoring over a small number of labeled posts—the scoring identifies when topic models are most effective at grouping labeled VAEM into a topic. The topic modeling scoring method is an important contribution of this study.

This study also presents detailed reporting, including comparisons, on a range of classification models, including traditional machine learning models and deep neural (deep learning) networks. Their effectiveness was measured against different-sized data sets, emulating data sizes that are likely to be available to other researchers [43], and we used charts (Multimedia Appendix 2) to illustrate how the amount of training data affects model recall and precision.

### Limitations

There are unavoidable issues and potential biases that result from using any social media data. A limitation of this study is the use of only English-language tweets as data source; the approach needs to be validated by using other social media data sources and other languages. Although the data collection for this study spanned a year and included some potential trend patterns during influenza seasons, a long-term data collection would be better for any analysis of trends. At the time of the study, a full year’s data were required to properly train and evaluate the classifiers—this was in part because of the limited pipeline of the Twitter application program interface and because data collection was from a period before the COVID-19 pandemic and signals were correspondingly less frequent compared with those found during the COVID-19 vaccines rollout.

However, the proposed VAEM-Mine method can identify VAEM with *F*_1_ score of 0.91 and is applicable to any similar problem of detecting personal health mentions in social media posts based on the language of conversations.

### Conclusions and Future Research

We have determined that the VAEM-Mine method is an effective approach for both identifying and applying the topic models and classifiers that, when combined, can filter out the vast amount of irrelevant vaccine-related conversations and isolate VAEMs.

A key contribution of this study is that appropriately scored topic modeling is highly effective for identifying social posts that might contain VAEM. The technique of *F*_1_ scoring of topic models based on a small number of labeled posts, identified in this study, is practical and easily implementable and can be used by other researchers to assist with identifying topic models that group texts on specific language features.

The volume of social media posts regarding the current COVID-19 pandemic is immense, but those that are related to personally experiencing illness owing to the virus or vaccines are a small portion of these; however, they contain similar language. Currently, we are applying the VAEM-Mine method to both internally gathered and published [44] COVID-19 vaccine–related Twitter data sets to examine trends in VAEM reporting. There are several ways in which the identified VAEM posts can be used for vaccine safety signal detection. Among them are (1) examining individual posts by domain experts; (2) further classifying the posts to identify adverse events of special interest, which include vascular, neurological, or allergic disorders and enhanced disease; and (3) measuring changes of post volumes that might indicate unfolding events.

This paper interprets the success of the VAEM-Mine method in terms of percentages of data captured by the method and compares classifiers in terms of *F*_1_ scores. Future studies can analyze the method’s success in terms of model explainability [45].

## Appendix

Multimedia Appendix 1 Model definitions and parameters.

Multimedia Appendix 2 Classification performance analysis.

Multimedia Appendix 3 Verification of best topic model.

## Abbreviations

AEFI: adverse events following immunization
CNN: Convolutional Neural Network
LSTM: Long Short-Term Memory
RoBERTa: Robustly Optimized Bidirectional Encoder Representations Pretraining Approach
VAEM: vaccine adverse event mention
