# Supplementary material for: Vaccine Adverse Event Mining of Twitter Conversations: 2-Phase Classification Study
Source: JMIR Med Inform. 2022 Jun 16;10(6):e34305. doi: 10.2196/34305 (PMC9247809; doi:10.2196/34305)
Supplement: Multimedia Appendix 1 [file medinform_v10i6e34305_app1.docx]

## Multimedia Appendix 1

Table 1 presents parameters and architectures of the classification models used in this study. For the traditional models, the vectorization method and parameters are also presented. For the traditional models, only the specific parameters the author used are presented. For the neural networks trained from scratch there are a few standard settings, such as optimizer and learning rate, which were used throughout. The Transformers were used with their defaults throughout, the key values from their configurations are presented. For all the neural networks, a lot of the experimentation was to assess the optimum number of epochs and minibatches to get the best from the model, which was usually just before the models started overfitting — and was assessed based on changes in validation loss, and - after training - on test F1-Scores. For the models that were trained from scratch, many experiments were conducted to arrive at these settings, but the detail of these is not presented.

The models were mostly trained on a PC with 16GB of RAM and a Nvidia GTX1080 graphics card, which has 8GB of RAM. The larger Transformers required a GPU with more RAM, so we used Google Colaboratory (Colab), which enabled the use of a Nvidia Tesla T4 with 15GB of RAM. An epoch of training on Colab typically took around 10 minutes. The number of epochs to convergence depended on the model and ranged from 3 epochs up to 12 epochs.

Table 1. Model definitions and parameters

| **Model** | **Model Definition** | **Vectorizer Definition** |
| --- | --- | --- |
| Logistic Regression CV | LogisticRegressionCV(  Cs=50,   max_iter=2000,   random_state=23 ) | TfidfVectorizer(  sublinear_tf=True,  max_df=0.5,   ngram_range = (1, 2),  use_idf=False ) |
| Stochastic Gradient Descent Classifier | SGDClassifier(  alpha=0.0001,   max_iter=50000,  penalty='l2',   tol=0.001,  random_state=23 ) | TfidfVectorizer(  sublinear_tf=True,  max_df=0.5,   token_pattern ='(?ui)\\b\\w*[A-Za-z]{2,}\\w*\\b',  ngram_range=(1, 2),  use_idf=True ) |
| Linear Support Vector Machines | LinearSVC(  C=1,   tol=0.001,   random_state=23 ) | TfidfVectorizer(  sublinear_tf=True,  binary=False,  ngram_range=(1, 2),  use_idf=True ) |
| Random Forest Classifier | RandomForestClassifier(  n_estimators=1000,   max_features=10,   max_depth=None,  min_samples_leaf=1,  min_samples_split=3,  criterion='entropy',   bootstrap=False,   oob_score=False,   random_state=23 ) | TfidfVectorizer(  max_df=0.5,  sublinear_tf=True,  token_pattern=u'(?ui)\\b\\w*[A-Za-z]{2,}\\w*\\b',  use_idf=False ) |
| Extra Trees Classifier | ExtraTreesClassifier(  n_estimators=1000,   max_features=10,   max_depth=None,  min_samples_leaf=1,  min_samples_split=3,  criterion='entropy',   bootstrap=False,   oob_score=False,   random_state=23 ) | TfidfVectorizer(  max_df=0.5,  sublinear_tf=True,  token_pattern=u'(?ui)\\b\\w*[A-Za-z]{2,}\\w*\\b',  use_idf=False ) |
| Multinomial Naïve Bayes | MultinomialNB(  alpha=0.15,   class_prior=None,   fit_prior=False ) | TfidfVectorizer(  sublinear_tf=True,  max_features = None,  max_df=0.5,   ngram_range = (1, 2),  token_pattern = '(?ui)\\b\\w*[A-Za-z]{2,}\\w*\\b',  use_idf=False ) |
| Naïve Bayes SVM | NBSVM(  C=1,   alpha = 0.01,   beta=1 ) | TfidfVectorizer(  sublinear_tf=True,  binary = False,  norm = 'l1',  max_features = 10000,   max_df=0.5,   ngram_range = (1, 3),  use_idf=False ) |
| XGBoost | XGBClassifier(  learning_rate=0.04,   n_estimators=900,   colsample_bytree=0.6,  gamma=1,  max_depth=5,  min_child_weight=1,  subsample=0.6,  objective='binary:logistic',  random_state=23 ) | TfidfVectorizer(  max_df=0.5,  sublinear_tf=True,  use_idf=True ) |
| All Neural Networks trained from scratch | activation : selu optimizer : AdamW learning_rate : 0.001 init_weight : True init_weight_value : 2.0 optim_momentum_value : 0.9 batch_normalizations : False clip : 5 weight_decay : 1e-8 batch_size : 32 | |
| CNN | [Conv2d(1, 100, kernel_size=(1, 100), stride=(1, 1), bias=False),   Conv2d(1, 100, kernel_size=(2, 100), stride=(1, 1), padding=(1, 0), bias=False),   Conv2d(1, 100, kernel_size=(3, 100), stride=(1, 1), padding=(1, 0), bias=False)] CNN_Text(  (embed): Embedding(4882, 100, padding_idx=1, scale_grad_by_freq=True)  (dropout): Dropout(p=0.5, inplace=False)  (dropout_embed): Dropout(p=0.1, inplace=False)  (fc): Linear(in_features=300, out_features=2, bias=True) ) | |
| CNN-BiLSTM | [Conv2d(1, 100, kernel_size=(1, 100), stride=(1, 1)),   Conv2d(1, 100, kernel_size=(2, 100), stride=(1, 1), padding=(1, 0)),   Conv2d(1, 100, kernel_size=(3, 100), stride=(1, 1), padding=(1, 0))] CNN_BiLSTM(  (embed): Embedding(4882, 100, padding_idx=1)  (bilstm): LSTM(100, 300, num_layers=2, dropout=0.5, bidirectional=True)  (hidden2label1): Linear(in_features=900, out_features=450, bias=True)  (hidden2label2): Linear(in_features=450, out_features=2, bias=True)  (dropout): Dropout(p=0.5, inplace=False) ) | |
| CNN-BiGRU | [Conv2d(1, 100, kernel_size=(1, 100), stride=(1, 1)),   Conv2d(1, 100, kernel_size=(2, 100), stride=(1, 1), padding=(1, 0)),   Conv2d(1, 100, kernel_size=(3, 100), stride=(1, 1), padding=(1, 0))] CNN_BiGRU(  (embed): Embedding(4882, 100, padding_idx=1)  (bigru): GRU(100, 300, num_layers=2, dropout=0.5, bidirectional=True)  (hidden2label1): Linear(in_features=900, out_features=450, bias=True)  (hidden2label2): Linear(in_features=450, out_features=2, bias=True)  (dropout): Dropout(p=0.5, inplace=False) ) | |
| CNN-LSTM | CNN_LSTM(  (embed): Embedding(4882, 100, padding_idx=1)  (dropout): Dropout(p=0.5, inplace=False)  (lstm): LSTM(100, 300, num_layers=2, dropout=0.5)  (hidden2label1): Linear(in_features=600, out_features=300, bias=True)  (hidden2label2): Linear(in_features=300, out_features=2, bias=True) ) | |
| LSTM | LSTM(  (embed): Embedding(4882, 100, padding_idx=1)  (lstm): LSTM(100, 300, num_layers=2, dropout=0.5)  (hidden2label): Linear(in_features=300, out_features=2, bias=True)  (dropout): Dropout(p=0.5, inplace=False)  (dropout_embed): Dropout(p=0.1, inplace=False) ) | |
| BiLSTM | LSTM(100, 150, bias=False, dropout=0.5, bidirectional=True) BiLSTM(  (embed): Embedding(4882, 100, padding_idx=1)  (bilstm): LSTM(100, 150, bias=False, dropout=0.5, bidirectional=True)  (hidden2label1): Linear(in_features=300, out_features=150, bias=True)  (hidden2label2): Linear(in_features=150, out_features=2, bias=True) ) | |
| GRU | GRU(  (embed): Embedding(4882, 100, padding_idx=1)  (gru): GRU(100, 300, num_layers=2, dropout=0.5)  (hidden2label): Linear(in_features=300, out_features=2, bias=True)  (dropout): Dropout(p=0.5, inplace=False) ) | |
| BiGRU | BiGRU(  (embed): Embedding(4882, 100, padding_idx=1)  (bigru): GRU(100, 300, num_layers=2, dropout=0.5, bidirectional=True)  (hidden2label): Linear(in_features=600, out_features=2, bias=True)  (dropout): Dropout(p=0.5, inplace=False) ) | |
| All Transformers | max_seq_length : 64  learning_rate : 2e-5  batch_size : 32, or 16 for larger models  iterations per epoch : length training data / batch size  adam_epsilon : 1e-8  warmup_steps : 0  max_grad_norm : 1.0  random_seed : 42 | |
| BERT | {  "attention_probs_dropout_prob": 0.1,  "hidden_act": "gelu",  "hidden_dropout_prob": 0.1,  "hidden_size": 768,  "initializer_range": 0.02,  "intermediate_size": 3072,  "layer_norm_eps": 1e-12,  "max_position_embeddings": 512,  "num_attention_heads": 12,  "num_hidden_layers": 12,  "output_attentions": false,  "output_hidden_states": false,  "output_past": true,  "type_vocab_size": 2,  "vocab_size": 30522 } | |
| RoBERTa | {  "attention_probs_dropout_prob": 0.1,  "hidden_act": "gelu",  "hidden_dropout_prob": 0.1,  "hidden_size": 768,  "initializer_range": 0.02,  "intermediate_size": 3072,  "layer_norm_eps": 1e-05,  "max_position_embeddings": 514,  "num_attention_heads": 12,  "num_hidden_layers": 12,  "output_attentions": false,  "output_hidden_states": false,  "output_past": true,  "type_vocab_size": 1,  "vocab_size": 50265 } | |
| RoBERTa Large | {  "attention_probs_dropout_prob": 0.1,  "hidden_act": "gelu",  "hidden_dropout_prob": 0.1,  "hidden_size": 1024,  "initializer_range": 0.02,  "intermediate_size": 4096,  "layer_norm_eps": 1e-05,  "max_position_embeddings": 514,  "num_attention_heads": 16,  "num_hidden_layers": 24,  "output_attentions": false,  "output_hidden_states": false,  "output_past": true,  "pruned_heads": {},  "torchscript": false,  "type_vocab_size": 1,  "vocab_size": 50265 } | |
| XLNet | {  "attn_type": "bi",  "clamp_len": -1,  "d_head": 64,  "d_inner": 3072,  "d_model": 768,  "dropout": 0.1,  "end_n_top": 5,  "ff_activation": "gelu",  "initializer_range": 0.02,  "layer_norm_eps": 1e-12,  "n_head": 12,  "n_layer": 12,  "n_token": 32000,  "output_attentions": false,  "output_hidden_states": false,  "output_past": true,  "start_n_top": 5,  "summary_activation": "tanh",  "summary_last_dropout": 0.1,  "summary_type": "last",  "summary_use_proj": true,  "untie_r": true, } | |
| XLNet Large | {  "clamp_len": -1,  "d_head": 64,  "d_inner": 4096,  "d_model": 1024,  "dropout": 0.1,  "end_n_top": 5,  "ff_activation": "gelu",  "initializer_range": 0.02,  "layer_norm_eps": 1e-12,  "n_head": 16,  "n_layer": 24,  "output_attentions": false,  "output_hidden_states": false,  "output_past": true,  "start_n_top": 5,  "summary_activation": "tanh",  "summary_last_dropout": 0.1,  "summary_type": "last",  "summary_use_proj": true,  "vocab_size": 32000 } | |
| XLM | {  "attention_dropout": 0.1,  "dropout": 0.1,  "emb_dim": 2048,  "init_std": 0.02,  "layer_norm_eps": 1e-12,  "mask_index": 5,  "max_position_embeddings": 512,  "n_heads": 16,  "n_layers": 12,  "output_attentions": false,  "output_hidden_states": false,  "output_past": true,  "pad_index": 2,  "start_n_top": 5,  "use_lang_emb": true,  "summary_first_dropout": 0.1,  "summary_proj_to_labels": true,  "summary_type": "first",  "summary_use_proj": true,  "vocab_size": 30145 } | |
