# Supplementary material for: Vaccine Adverse Event Mining of Twitter Conversations: 2-Phase Classification Study
Source: JMIR Med Inform. 2022 Jun 16;10(6):e34305. doi: 10.2196/34305 (PMC9247809; doi:10.2196/34305)
Supplement: Multimedia Appendix 2 [file medinform_v10i6e34305_app2.docx]

## Multimedia Appendix 2

Models trained on the initially collected data were retrained on a larger amount of data, and having more data enabled optimal training of the larger Transformer-based models. The following analysis examines how the models performed with these two training phases.

### Imbalanced Test data with Phase-One models

The Phase-One Test dataset was highly imbalanced, having only 90 VAEM vs. 524 non-VAEM – but it was used throughout as a standard because it represented a real scenario of tweets that had been identified as belonging to our geographical region. During our initial, Phase-One testing, we noted that the Imbalanced Test dataset suited models that favoured the non-VAEM (negative) class. That is, models that tended to shift both false and true positives into the negative class did well with this test set. For instance, the Naïve Bayes SVM model eliminated many false positives and thereby achieved the highest precision among the traditional models, but it also eliminated true positives and so had the poorest recall. However, it was awarded the highest F1 Score among the traditional models just because there was a much high number of the non-VAEM class in the test data. That is, the model’s precision benefitted by removing many false positives, which offset the penalty due to its removing (somewhat fewer) true positives. Because our requirement was to identify as much VAEM as possible with relative precision, we needed to favour VAEM recall over precision; therefore, we also tested with an F1-Beta score, using a beta of 1.3. We observed that the Naïve Bayes SVM F1-Beta score was then closer to the middle of the scoring range, and that BERT, with its relatively higher recall, was promoted to second place – see Figure 1.

Figure 1. Phase-One models and Imbalanced Test data - F1 vs F1 Beta
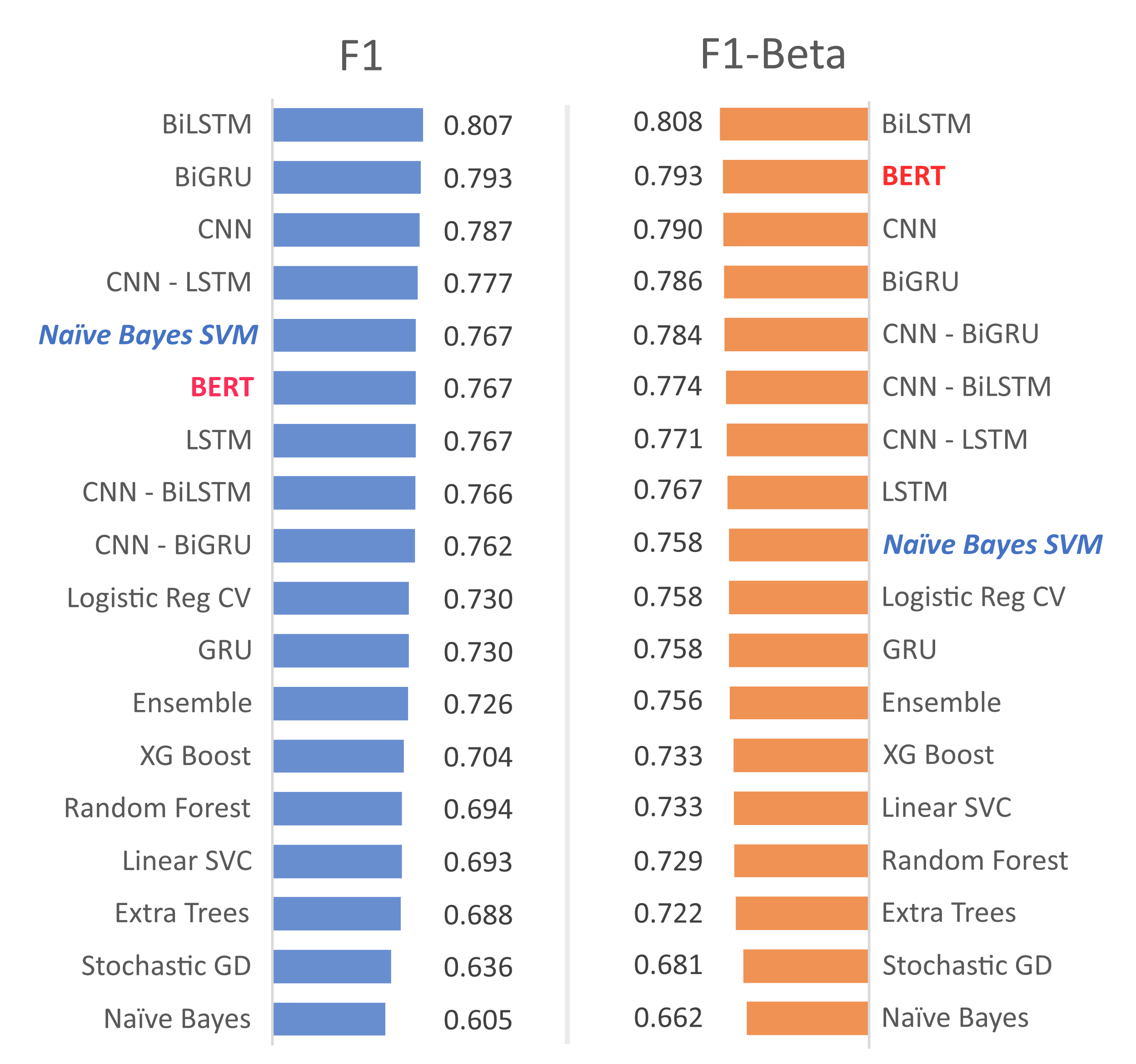


The relationships between F1 and F1-Beta, together with the variations of precision and recall are depicted in Figure 2. The models are displayed in increasing order of their F1-Beta scores. Note that Naïve Bayes SVM has one of the highest precision values, but also the lowest recall, and so is penalised by the F1-Beta score. Conversely, the BERT model has a high recall but a relatively lower precision, resulting in the same F1 Score (0.767) as the Naïve Bayes SVM model – but that because of its recall is favoured by the F1-Beta score. The chart shows that the traditional classifiers tend to have a higher recall but with poor precision, but that after the point where the Naïve Bayes SVM enters the chart, the remainder of the models (which are based on neural networks) have precision and recall values that are closer to each other.

Figure 2. Phase-One models on Imbalanced Test data - F1 Scores and their measures


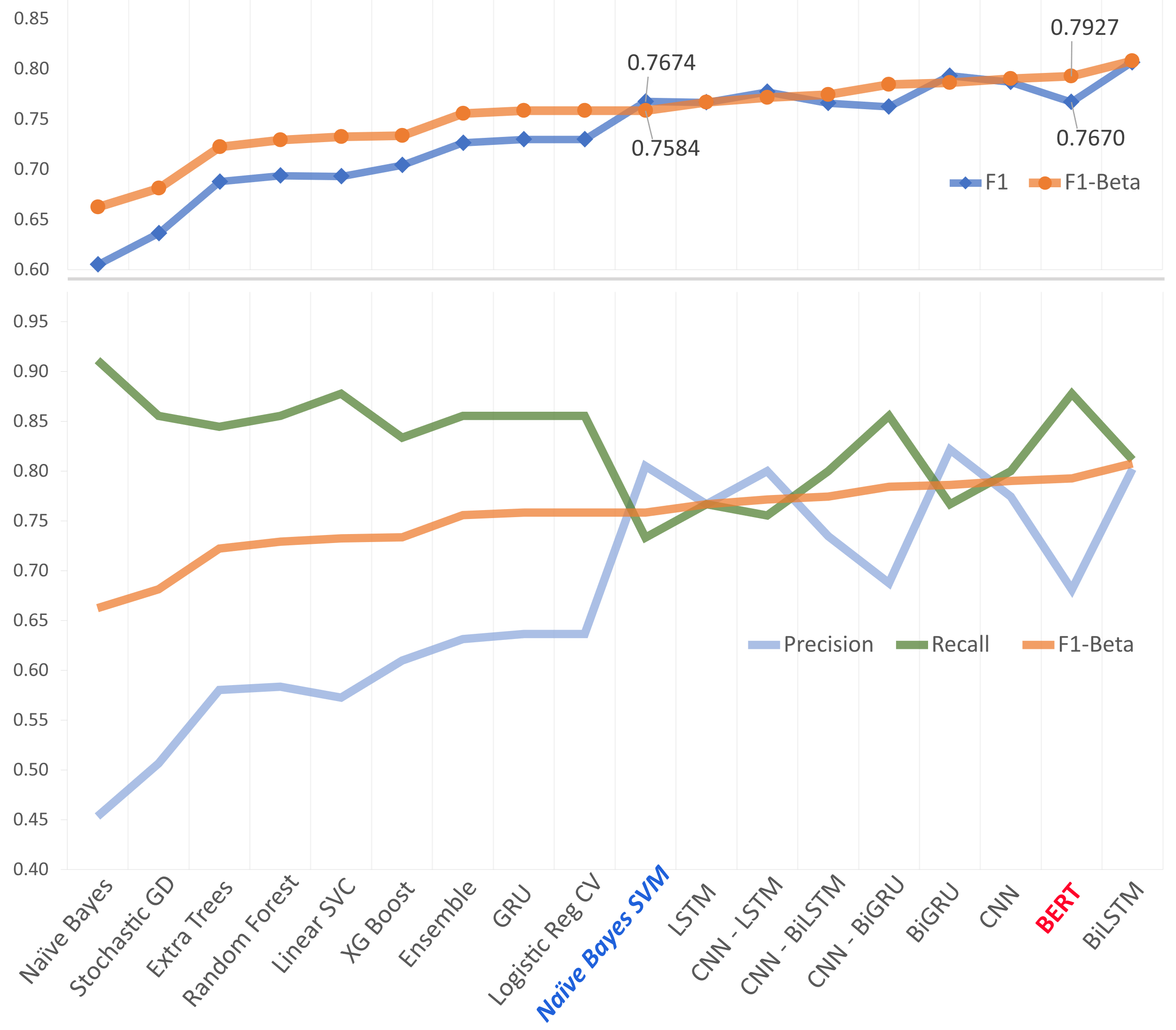


### Imbalanced Test data with Phase-Two models

Almost all the models’ F1 scores increased by at least 5%, after the models were re-trained with the additional data available in the second phase of training (where the training data increased by over 5 times from 3,519 records to 19,249). The Phase-Two classifiers included five new Transformer models. See Figure 3 for the performance measures against the Imbalanced Test data, which again show some extreme differences in precision and recall, but significantly, fewer examples of a great divergence between precision and recall among the lower-order models, with F1 and F1-Beta being more aligned. The upwards trajectory of the resulting F1 scores is rather steeper than it was with the Phase-One models – there is a 20% difference between the worst and best performing models. This is due to the top performing Transformer models having a 10% better performance than the top performing traditional models, and effectively coping with the imbalanced data – the “Balanced Test data with Phase-Two models” section shows that they could obtain very similar F1 scores on this data as they did on the balanced Test data. The RoBERTa Large model achieved an F1 score of 0.919, and an F1-Beta of 0.923.

Figure 3. Phase-Two models on Imbalanced Test data - F1 Scores and their measures


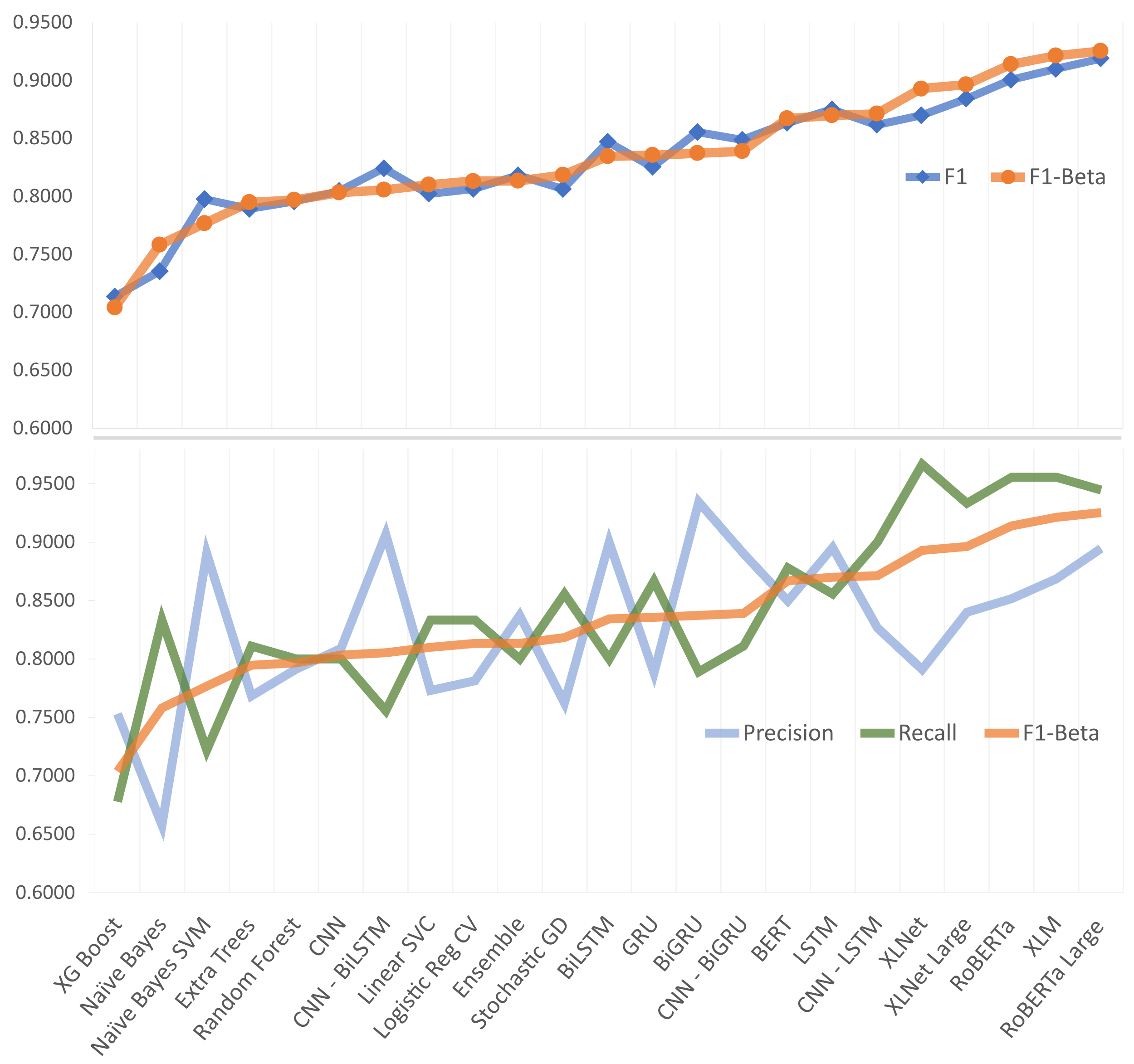


### Balanced Test data with Phase-One models

The Balanced Test dataset consisted of 828 records with 431 VAEM and 397 non-VAEM. The behaviour of the models when tested with this data was a lot more regular, even with the Phase-One models (Figure 4).

Figure 4. Phase-One models on Balanced Test data - F1 Scores and their measures


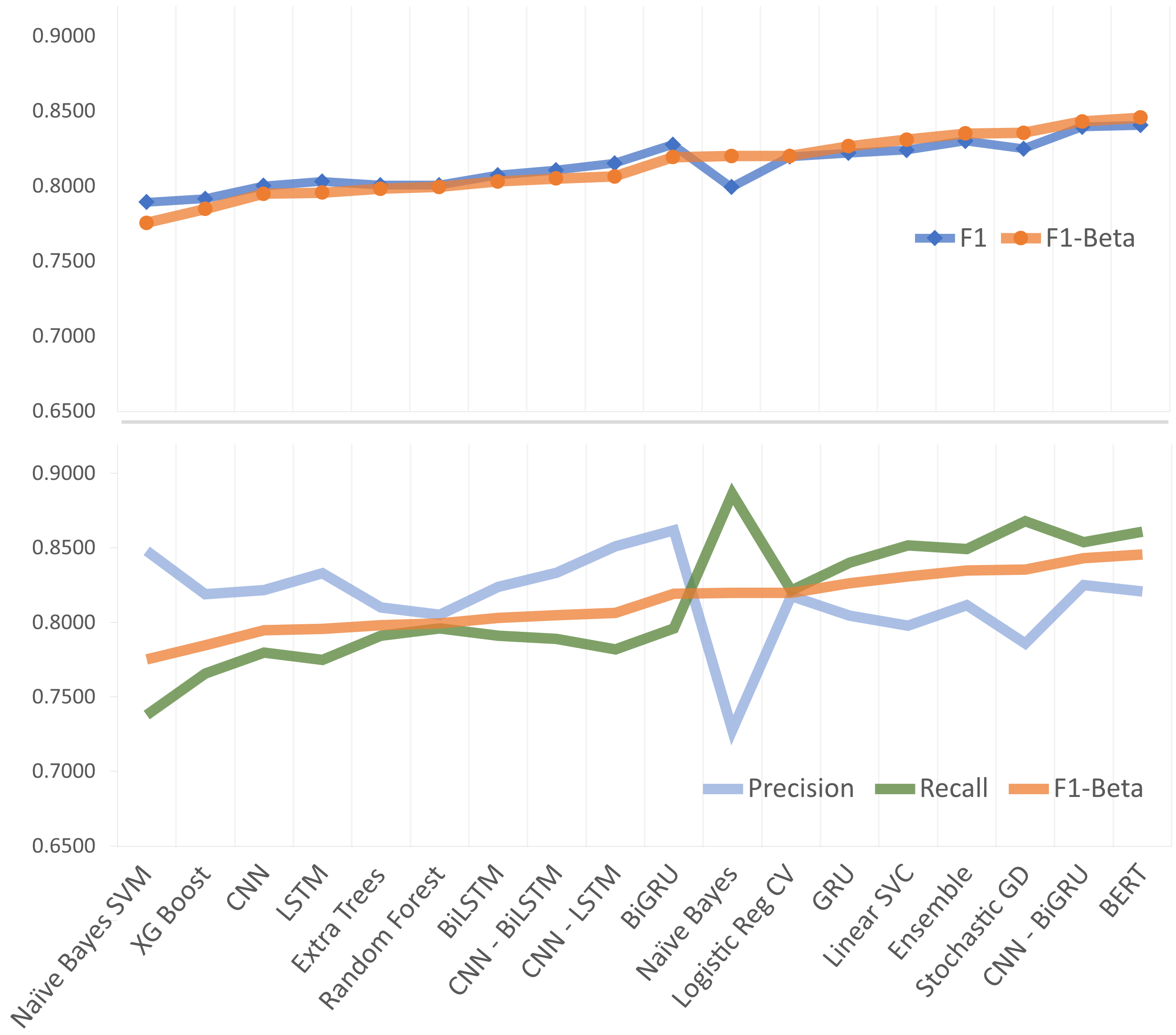


Precision initially exceeded recall, then switched to recall exceeding precision, but with a more consistent relationship when compared with the evaluations on the Imbalanced Test data. The Naïve Bayes-based models are noteworthy: In this scenario, Naïve Bayes SVM was the poorest performer, and standard Naïve Bayes scored much better – the model’s very high recall was weighted by the F1-Beta calculation to offset its poor precision and give it a score in the middle of the range. BERT and CNN-BiGRU were the best models – they both had combinations of high recall and precision, but the Ensemble of traditional models had a similar balance of precision and recall and was the fourth best model. Testing on the Balanced Test data did not show such a clear distinction between the performance of the traditional models and neural networks, several traditional classifiers performed better than some of the neural networks.

### Balanced Test data with Phase-Two models

As previously noted, the models’ F1 scores increased by at least 5% after the models were re-trained with the larger data, and when tested with the balanced Test data their performance improved compared to that when tested with the Imbalanced Test dataset, all the F1 scores were above 0.8 (Figure 5).

Figure 5. Phase-Two models on Balanced Test data - F1 Scores and their measures


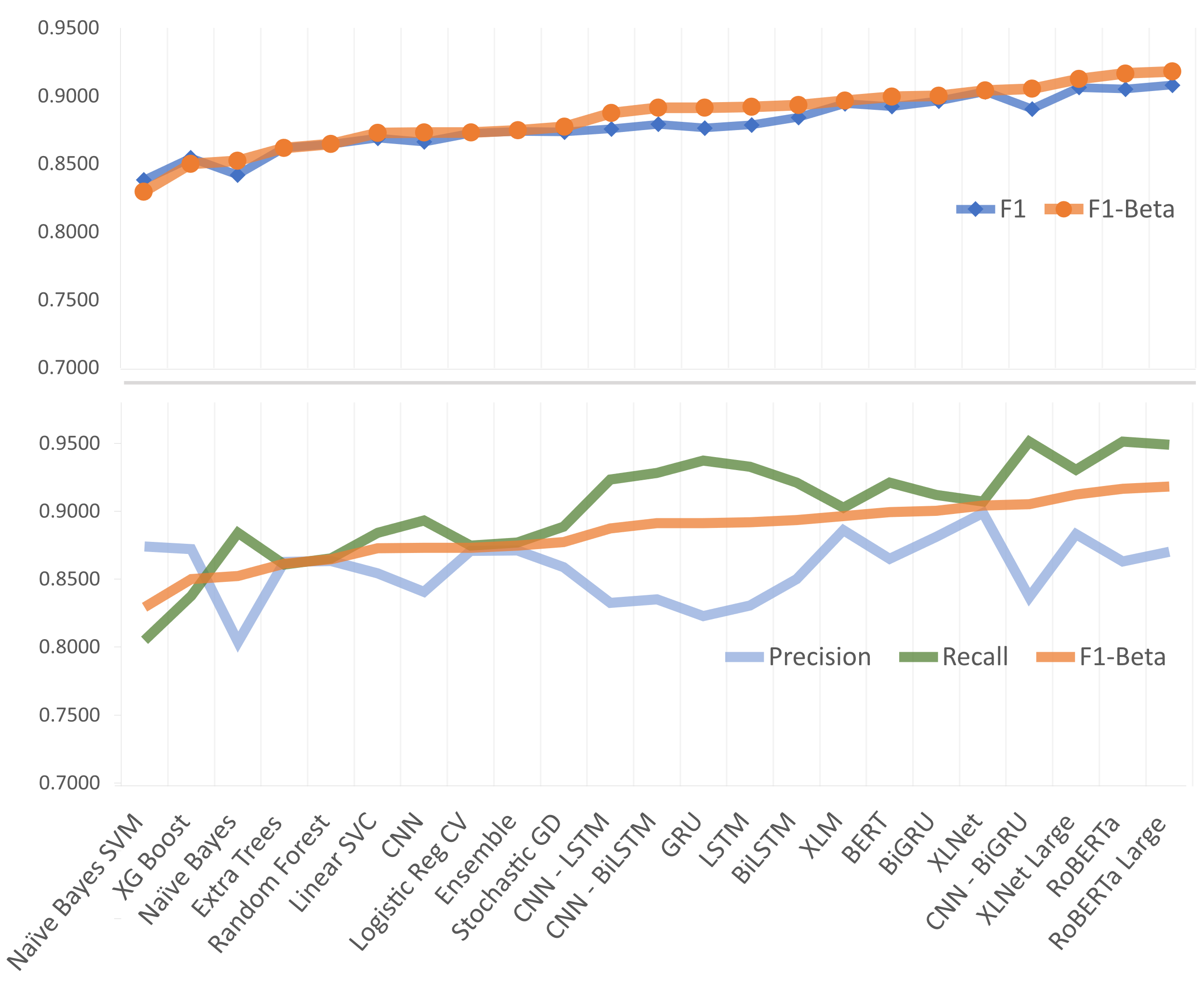


The Phase-Two results depicted in Figure 5 show an even closer relationship between the F1 and F1-Beta scores, with a clear progression in scores from the traditional models to the neural network-based models – there are no longer traditional models’ results interspersed within those from the neural networks models. The RoBERTa Large model achieved an F1 score of 0.908 and an F1-Beta of 0.918 – as indicated earlier these align with the scores achieved on the Imbalanced Test data, so we conclude that training on the larger dataset has achieved an optimal result from the model.

### Phase-Two models vs Phase-One models

Figure 6 shows the relative performance of the Phase-Two models vs the Phase-One models when evaluated with the Balanced Test data – as such they represent the best performances of both training phases. There are five extra entries for the Transformer models added to the Phase-Two models. Note that the highest scores from the Phase-One trained models shown in the bottom part of the chart are around 0.85, whereas only the bottom three Phase-Two trained models are on or below 0.85; that almost all scores have increased by at least 5%; and that there is a greater overall rate of improvement noticeable in the slope of the Phase-Two trained models.

Figure 6. Phase-Two vs Phase-One models - F1 scores on Balanced Test data


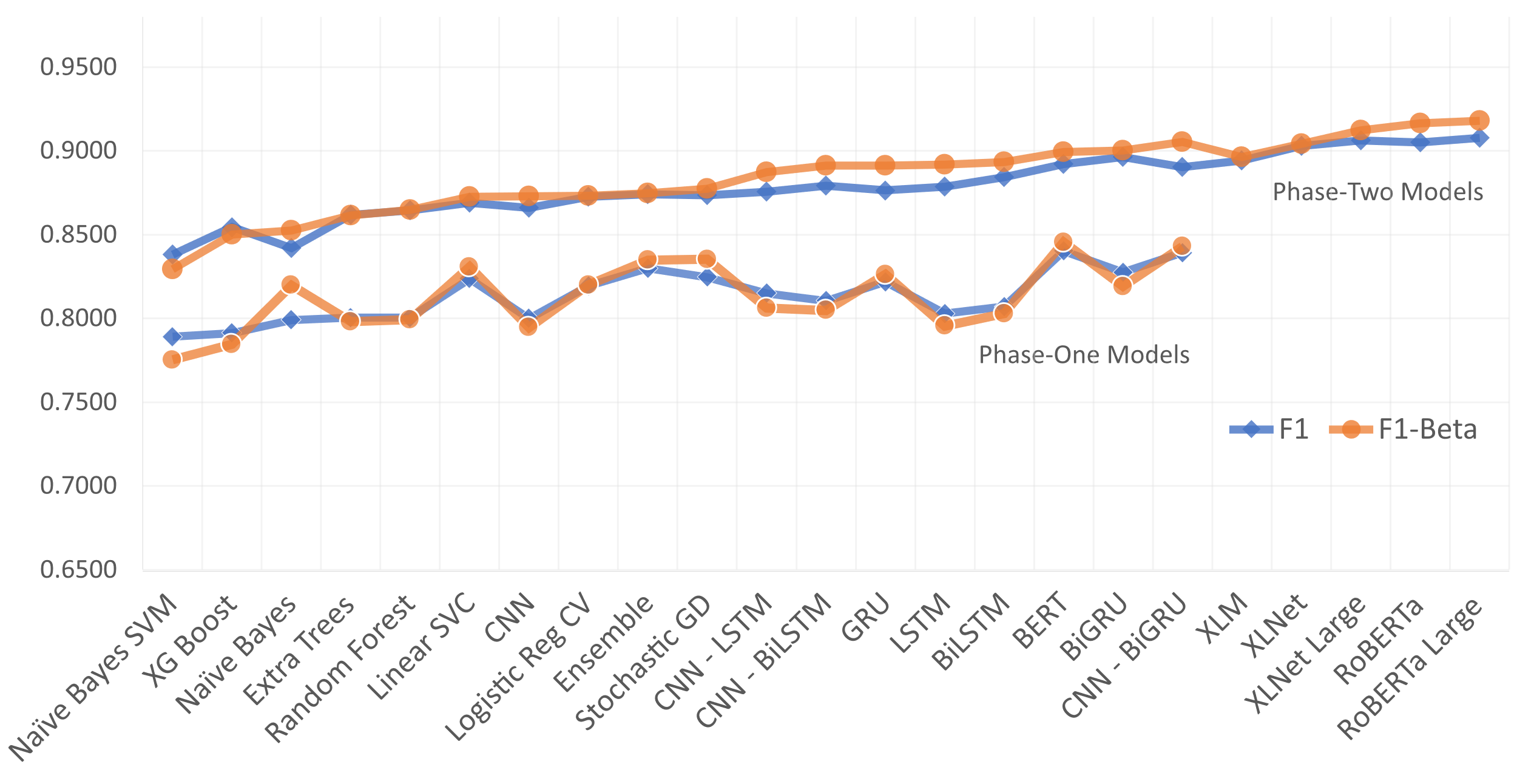


To conclude, the descriptions of classification results and the analysis presented above should assist other researchers to make decisions about classifiers and the amount of data required, should they be encountering similar texts and volumes of data. The VAEM-Mine method’s design incorporates an evaluation of the classification process with the decision point that allows for collection of more data. After retraining the models on 19 thousand records and evaluating with a larger balanced test dataset we observed significant performance gains and the ability to use more powerful classifiers, with the Transformer models proving to be most capable. The F1 scores of the Roberta Large model are exemplary and have established that classification very effectively isolates the VAEM from the incoming data (which was the output of the topic modelling component of the VAEM-Mine method).
