# Supplementary material for: Vaccine Adverse Event Mining of Twitter Conversations: 2-Phase Classification Study
Source: JMIR Med Inform. 2022 Jun 16;10(6):e34305. doi: 10.2196/34305 (PMC9247809; doi:10.2196/34305)
Supplement: Multimedia Appendix 3 [file medinform_v10i6e34305_app3.docx]

## Multimedia Appendix 3

As previously described, topic modelling was used to initially filter tweets, obtaining a cohort of tweets most likely to contain VAEM. The topic model used was a 14-topic Dirichlet Multinomial Mixture (DMM) [1], and topic 13 of that model was identified as the best topic by the F1-based scoring system. The Topic 13 tweets were subsequently labelled for classification purposes as either VAEM or not - and used for the ongoing work. The remaining thirteen topics of the 14-topic model were not labelled and were not thereafter used, and it was unknown whether these might contain VAEM.

To verify that it had been justified to discard topics other than Topic 13 as unlikely to contain VAEM, ten thousand samples were taken of the *total* data of the 14-topic model (i.e., inclusive of the already labelled Topic 13), and the as-yet unlabelled tweets were labelled - as either VAEM or not. The result was 10 samples, each consisting of 10 randomly sampled groups of 100 records and combined into 1,000-record samples, for a total of 10,000 sampled records. Only one VAEM record emerged in another topic apart from Topic 13. This information is presented in Figure *1*. For simplicity, only topic numbers containing VAEM are assigned a separate bar in the chart, all other topics are combined into the third “All Others” bar. Within topics 13 and 1 (“Thirteen” and “One” on the chart) the VAEM containing tweets are depicted by the darker portions on the right of each bar, the lighter portion to the left is non-VAEM. These are counted, for instance in the Topic 13 sample there are 127 VAEM and 1,564 non-VAEM, a total of 1,691. The percentage label to the right of the bar for Topic 13 indicates the proportion of all VAEM in the topic, which is 99.2%. That is, 127 of the total 128 VAEM in the samples are found in Topic 13, and therefore only 0.8% of VAEM are found in other topics. Topic 13 consists of only 16.9% of the total sampled data, which is consistent with the filtering effect observed during the topic modelling.

Figure 1. Sample Distribution of VAEM in topics


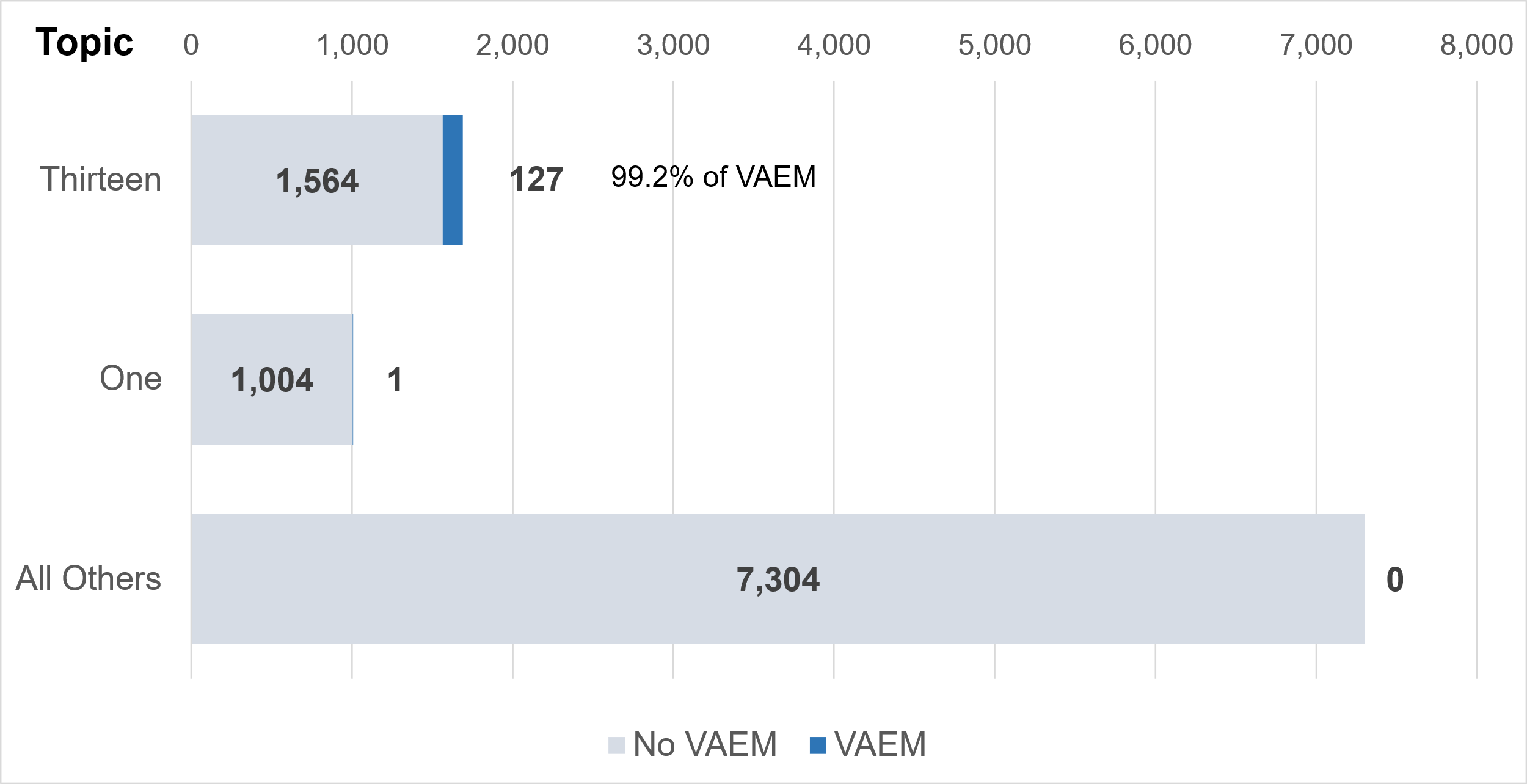


Five additional 1,000 sized samples were taken of topics *excluding* Topic 13, only 2 VAEM were found in the 5,000 samples, which was 0.04% of the data. These numbers verify the effectiveness of Topic 13 of the 14-topic DMM model for filtering vaccine adverse event mentions. To be consistent with the samples and allowing for error it is estimated that 99% of the vaccine adverse event mention data is being included in records collected by Topic 13 of the DMM 14-topic model.

References:

1. Nguyen DQ. jLDADMM: A Java package for the LDA and DMM topic models. 2018;(Dmm):1–5. Available from: http://arxiv.org/abs/1808.03835
